# Burden of Proof: The Debate Surrounding Aerotoxic Syndrome

**DOI:** 10.1177/00220094221074819

**Published:** 2022-01-28

**Authors:** Stephen E. Mawdsley

**Affiliations:** 1980 University of Bristol, UK

**Keywords:** Aerotoxic, aircrew, biomedicine, jet, labor activism, occupational health

## Abstract

Since the 1980s, some commercial airline pilots and flight crews in the United States, United Kingdom and Australia began to report an illness they believed was caused by exposure to contaminated cabin air. Despite a body of scientific research and health activism calling for this condition, termed Aerotoxic Syndrome (AS), to be classified an occupational illness, it has not been accepted as a clinical entity because its causation remains contested. This article contends that debates over the recognition of AS have been shaped by the politics of science and what can be considered evidence of a causal link; the burden of proof lay with survivors and their allies rather than with airlines and manufacturers. The history of AS shows the challenges of reacting to health risks in a global industry that provides an important form of transportation, and enjoys considerable political and economic influence. It also reveals that at the heart of commercial jet air travel remains an unresolved public health issue, and those who claim to be suffering from AS expected prompt recognition, reform and assistance in light of scientific research and personal testimony, as well as a range of chemical, medical, legal and air safety reports.

In 2008, flight attendant Matthew Bass began to feel unwell.^1^ Although physically active and in good health, he started to lose weight, contend with bouts of exhaustion, and experience digestive issues. ‘There was a slight tremor in his hands’, his father remembered. ‘I asked Matthew about it and he said it was nothing and said it was probably tiredness’.^2^ In an effort to identify the cause of his malaise, Bass underwent medical tests, but the results were inconclusive. Without a diagnosis, he assumed that he was suffering from Crohn's Disease, an inflammation of the bowels. Bass’ health continued to decline and on 30 January 2014, after relaxing over drinks with friends, he lay down and never regained consciousness. An investigation was undertaken into his death, but it did not reveal the cause of his longstanding malady. His parents paid for a comprehensive post-mortem, which showed evidence of ‘chronic exposure to organophosphates’.^3^ They believed their son's slow deterioration was due to his exposure to contaminated cabin air while working as a flight attendant. ‘The most difficult part of all of this to come to terms with’, Bass’ father reflected, ‘is that the airlines, industry and the government have known this has been happening for decades but are doing nothing to prevent it’.^4^ Did Bass’ occupation expose him to toxic substances? What was known about the potential health risks of contaminated cabin air, and could it be linked to a disease?

Aerotoxic Syndrome (AS) was coined in 1999 to describe an illness that some believed was caused by contaminated cabin air in jet aircraft. Despite a growing body of peer-reviewed research and health activism calling for AS to be classified an occupational illness, it has not been recognized as a clinical entity because its causation remains disputed.^5^ Proponents of AS, including many pilots, flight attendants, and their unions, gathered evidence and pressed employers to recognize the health risks of contaminated cabin air. In opposition, aircraft manufacturers and airlines have challenged the evidence, and cited the confidentiality of personnel health files and the potential impact of contributing risk factors, such as genetics and environment, as grounds for dissent.^6^

Drawing on newspapers, testimonies and scientific studies, this article argues that debates over AS have been shaped by the politics of science and a protracted battle over what constituted sufficient proof of causation.^7^ As the burden of proof lay with survivors and their allies, scientific evidence was needed before AS could be formally recognized.^8^ The history of AS reveals the challenges of identifying and responding to health risks in a globalized industry providing key transportation infrastructure and enjoying considerable political and economic influence. It also shows that at the heart of commercial air travel remains an unresolved public health issue, and those who claim to be its victims expected prompt government intervention, recognition and medical treatments in response to research studies and personal testimony supporting AS, as well as a range of alarming chemical, medical, legal and air safety reports.

This historical study of AS speaks to scholarship at the intersection of biomedicine, labour activism and public health.^9^ Historians of biomedicine have examined the politics of science, as well as the significance of research and the ethical challenges faced by scientists.^10^ In turn, labour historians have explored the relationship between employers and unions, and the improvements brought about through collective action.^11^ Finally, public health historians have shed light on the risks of cigarette smoke, coal dust and asbestos, and the activism of survivors and their allies to seek compensation and policy changes.^12^ This article offers a new case study to this body of historical literature by tracing how concerns about contaminated cabin air were approached and debated from the 1950s up to the recent past.

Since the 1950s, commercial air travel has eclipsed other forms of transportation and created new careers and trade opportunities. Airline advertisements have depicted such travel as safe, expedient, economical and comfortable.^13^ Part of this comfort was credited to the environmental control systems that deliver oxygenated air and recreate the feeling of an indoor atmosphere. However, cabin air is different from conventional indoor air because of the extreme external environment, high occupant density and dependence on technology.^14^ The high speed and altitude, as well as low pressure and temperature require cabin air to be drawn from outside the aircraft where it is heated, pressurized, circulated and eventually filtered.^15^

Decisions made by aircraft manufacturers in the early years of the industry had a lasting impact on the design of air intake systems. Some of the first passenger jets, including the Vickers VC10, McDonnell Douglas DC8 and Boeing 707, mounted air intakes for cabin air outside the jet engine.^16^ However, by the mid-1950s manufacturers began to favour a competing design that drew cabin air from within the compression chamber of the engine, known as bleed air.^17^ Although the bleed air design offered important economic, weight and aerodynamic benefits, it also increased the risk of exposing cabin air to the engine environment, which depending on conditions, could contain pyrolyzed (burnt) jet engine lubricant.^18^

Jet engine lubricant is a complex formulation of several different chemicals designed to withstand high temperature and pressure to protect moving engine parts. Some additives are known to be hazardous and have been the subject of historical events and warnings. The anti-wear agent TCP (tricresylphosphate), for instance, was first implicated in the 1930 outbreak of Jamaica Ginger Paralysis when some manufacturers adulterated the patent medicine Jamaica Ginger with TCP in a misguided attempt to circumvent rudimentary US government testing during National Prohibition. TCP was discovered to be a neurotoxin and consumption of the adulterated Jamaica Ginger caused a distinct form of paralysis, affecting over 50,000 people.^19^ Similarly, a decade before Rachel Carson published *Silent Spring* (1962), environmental researcher Solly Zuckerman released a report for the UK Minister of Agriculture in 1951 on the use of toxic chemicals in agriculture.^20^ The active ingredient in the popular pesticide Parathion (also known as Folidol) was an organophosphate similar in chemical composition to TCP. Zuckerman's report advised against the use of such pesticides due to their toxicity. As jet engine lubricant contained several chemicals, often including TCP, the potential health risks related to exposure in cabin air became central to debates over AS.

During the 1980s, some aircrews in the United States, United Kingdom and Australia, began to report an illness they believed were associated with inhaling pyrolyzed engine lubricant. These so-called fume events ranged from hazy smoke entering the cabin to the smell of old socks or burnt rubber.^21^ According to one industry memo: ‘Some of the events have been significant, in that the crew reported blue smoke with defined waves in the smoke’.^22^ Exposure to fumes could cause itchy eyes and nausea to more severe symptoms, such as loss of cognizant reasoning, tremors and blackouts.^23^ Pilots faced an elevated risk because aircraft ventilation designs prioritized cockpit air.^24^ Although fume events were initially presumed to be rare, reports from the 2000s indicated an incidence rate from as high as 1 in every 66 flights (1.5%) to as low as 1 in every 2000 flights (0.05%).^25^ Certain airlines experienced a higher incidence rate than others, and particular aircraft were especially susceptible.^26^

Concerns about the quality of cabin air began to be raised as larger jet aircraft entered service during the Cold War. Boeing Airplane Company was among the first manufacturers to study fume events in relation to the bleed air design. In 1953, aircrews serving on Boeing B-52 strategic bombers complained of the ‘presence of smoke and odor in the occupied compartments’.^27^ Boeing investigated the ‘air contamination problem’ with the assistance of the Bureau of Mines and the US military's research facility Edgewood Arsenal. Scientists conducted a series of engine tests to determine the chemistry of the aerosols in cabin air and the utility of installing air filtration systems. While their studies did not assess the ‘toxic effect of the contamination’, they showed that fume events occurred and might be reduced through ‘relocation of the bleed port’.^28^ Although some aircraft manufacturers showed concern about the bleed air design, they did not believe it was sufficiently dangerous to abandon.

The United States Air Force (USAF) also examined the problem of contaminated cabin air. In 1955, the Martin RB-57A (Canberra), a twin-engine jet primarily used for photo reconnaissance, was singled-out for scrutiny.^29^ Following a serious crash and casualties involving two RB-57As at a base in South Carolina, pilots later described how inhaling cabin air before the crash had led to a range of symptoms. ‘After being airborne approximately 45 minutes’, explained one senior pilot, ‘I became sick (metallic taste) to stomach with dryness of mouth, throat and stomach. Pressurization was turned off and clear vision panel opened and I immediately began feeling better’.^30^ After breathing cockpit air, another pilot reportedly ‘experienced blurred vision, became nauseated and experienced considerable dizziness’ until the cabin was ventilated.^31^ Such testimonies concerned USAF officials, as they were interested in keeping pilots safe and able to complete their missions using the latest jet aircraft. The alarming reports warranted further research and toxicology studies.

The USAF turned to pharmacologist Dr Stephen Krop and USAF Captain Ted Loomis to work with the Army Chemical Corps and Martin Aircraft Company to investigate ‘the effects of the cabin air in RB-57A aircraft on personnel’.^32^ As part of the study, Krop and Loomis considered whether ‘psychogenic’ factors and any ‘existing medical’ conditions affected the pilots. Forty-two USAF volunteers were recruited to inhale ‘the air from the air conditioning system of the aircraft’ in various settings. One test required participants to sit in gas chambers and inhale air sprayed with jet engine lubricant.^33^ Before the establishment of Institutional Review Boards, many American researchers were aware of medical ethics, but rarely motivated to apply them in practice.^34^ Krop and Loomis later acknowledged the health risks faced by volunteers and their means of mitigation. ‘Because of previous knowledge gained from animal experiments that thermal break-down products from the engine lubricant can lead to pathological changes in the lungs of animals, these experiments were approached with considerable caution’, they wrote.^35^ Although research ethics suffered under these circumstances, such tests were not unusual at the time and deemed essential to reduce the risks carried by others.^36^

Krop and Loomis’ final report on cabin air pointed to a health risk. Although they reasoned that bleed air was normally ‘safe for humans’, they also showed that there were potential risks ‘if the engine air was contaminated with sufficient lubricant’ as ‘untoward symptoms appeared in subjects exposed to this air’. Recorded symptoms included ‘eye, nasal and pharyngeal [throat] irritations’ as well as ‘nausea’ and ‘tightness in the chest’. The researchers determined that cabin air became contaminated because ‘the mechanical arrangement of the apparatus supplying engine air to the cabin presents the possibility of the air becoming contaminated with engine lubricant’.^37^ Evidence suggests that military officials determined the risk could be reduced if pilots used a personal breathing apparatus during flight and aircraft maintenance teams devoted special attention to engine seals. At a time of rapid investment in and reliance on jet aircraft for strategic military purposes, such decision making appeared pragmatic for USAF officials.^38^ Although this federally funded research revealed early concern for contaminated cabin air, the focus and mitigations involved short-term exposure in a military context and not long-term exposure in a commercial setting.

Despite early military and manufacturer investigations into contaminated cabin air, the issue was not actively pursued in the intervening decades as commercial airlines turned attention to route expansion and passenger comfort. While regulatory standards required aircrew to be notified of unsafe operating environments, achieving this condition in practice proved challenging.^39^ Prior to the 1980s, tobacco smoke on airlines was a major source of air pollution, and it was not until the enactment of smoking bans in the late 1980s and early 1990s that other contaminants in the cabin could be investigated.^40^ In addition, early toxicology studies on cabin air were varied in their sampling methods and not easy to incorporate into larger studies.^41^ This was further exacerbated by the limits of air monitoring equipment, which were primarily designed to measure humidity, carbon dioxide and ozone, rather than the complex toxicity of fume events.^42^

Reporting problems also concealed the extent of cabin air contamination. According to sources, some flight crews were reluctant to report cabin smoke out of concern for their jobs or a lack of knowledge about the issue.^43^ In addition, some airlines did not always pass reports about fume events on to regulators.^44^ Karsten Severin of the German Aircraft Accident Investigation Branch concluded in 2009 that: ‘I do have to conclude that we do not receive reports on all events’.^45^ The frequency of fume events remained underreported because the data was ‘considered proprietary by the airlines’ and ‘not made available’.^46^ Meetings held between flight crews and employers about incidents were often conducted in private, and in cases where crews won compensation claims for illness, evidence suggests that some may have signed non-disclosure agreements.^47^ These factors reduced awareness of AS and made the process of gathering evidence about fume events more difficult.

A lack of information about fume events meant that physicians were unprepared to help patients. Most physicians attending to pilots or flight attendants complaining of AS were uncertain about the cause or what tests to perform.^48^ According to physician Dr Chris Winder, even short-term exposure to toxic chemicals from jet engine lubricants may lead to a wide range of symptoms.^49^ Furthermore, clinical judgements may have been influenced by the airline industry. Physician Dr Mark Donohoe, who treated patients who believed they had AS, reported being approached by industry physicians and asked to reconsider his clinical diagnoses.^50^ Some researchers expressed concern that a corporate affiliation bias inspired industry physicians to challenge those who raised concerns.^51^ The combination of complex symptoms, a lack of diagnostic criteria and the potential for industry pressure appear to have limited wider clinical discussions.

As debates over AS lingered into the 2000s, pilots and aircrews expressed disappointment and frustration. ‘There has been a significant exercise in semantic tap-dancing by the regulatory authority’, one representative of Flight Attendants Association of Australia observed, ‘over whether this is a health issue or a safety issue as though there is some need for distinction between the two’. This observation revealed the challenges faced by AS proponents as they sought recognition for their illness. ‘If flight attendants are having to be carted off aircraft in wheelchairs and placed onto oxygen during descent, the health of these flight attendants has been affected to the extent where the safety of the flight and of those passengers has been compromised. Consequently, the issues of health and safety are not separate, but are inextricably intertwined’.^52^

A fuller understanding of AS has been stymied by coordinated opposition. AS opponents, including airlines and industry representatives, mobilized a range of strategies since the 2000s to challenge peer-reviewed research and the testimony of pilots and flight crews. Among the earliest approaches undertaken by industry representatives was to cite steady advances in aviation safety as proof of a strong commitment to public health. The former chief medical officer of American Airlines and consultant to manufacturer Airbus, Prof Michael Bagshaw, argued that the industry valued customer safety and that such respect was well earned. ‘If people believed that airlines were harming people deliberately and not taking every precaution’, Bagshaw stated during an interview, ‘people wouldn't fly with them’.^53^

Industry representatives also rejected the claim that fume events contained toxic chemicals that could cause illness. In 2000, Bruce Jones of British Aerospace reasoned that ‘we have no direct information on the clinical nature or cause of any individual symptoms’.^54^ Similarly, Bagshaw discounted any correlation. ‘I accept there are people suffering from some symptoms and signs’, he acknowledged, ‘whatever the origin’.^55^ He posited that there were perhaps other factors involved. ‘I accept that there are people who are unwell and these people's health may well be being harmed by breathing in fumes, but there is no independent scientific evidence of that at the moment’.^56^ When asked why research on contaminated air did not exist, Bagshaw pointed to technological limitations. ‘There is no way of monitoring the air at the moment’, he explained. ‘A lot of money is being spent at the moment to develop monitors. The monitors do not exist at the moment’.^57^ For some AS opponents, technical limits, as well as environmental and personal issues made it difficult to prove causation.

Industry representatives also questioned the methods and accuracy of toxicity samples gathered by researchers. In one case, they claimed that past studies using swab samples taken from cabin surfaces did not correlate to their presence in cabin air.^58^ ‘The components released into the passenger cabin during air-quality incidents and their possible concentrations cannot be determined from the experiments’, one representative asserted.^59^ In turn, British Aerospace representative Ivor Williams reflected that ‘what we are proud of is the fact that the contaminants that they found in the system are incredibly low, way below the maximum levels that are permitted by the authorities. They compare very favourably with WorkSafe and occupational health and safety levels’.^60^ For such industry representatives, evidence of toxicity was insufficient to justify intervention or alarm.

The frequency of fume events was also questioned. During a 1999 hearing, one airline official claimed that ‘log reports showed an odour was reported once in every 66 flights’ and that number appeared to be falling to ‘one odour occurrence in every 160 flights’.^61^ Similarly, British Aerospace claimed ‘reports of cabin air odours have been received from time and time and have predominantly been determined to be due to minor systems failures such as leaks from oil seals on aircraft engines’.^62^ By downplaying the frequency of fume events, AS opponents reinforced the assumption that they were an unlikely source of illness.

Although jet engine lubricant contained toxic chemicals, some suppliers offered testimony that they did not pose a health risk. Julian Plummer, Manager of Aviation Lubricant Sales with Mobil Oil Company, explained in a 2000 hearing that ‘Mobil do not consider accidental exposure to oil vapours in an aircraft cabin to be ‘normal use’, but the levels that can be reached are comprehended by our internal and published risk assessments and are considered safe’.^63^ The company explained that ‘we do not believe that any of the symptoms, reported by individuals claiming to have been exposed to mists or odours of Mobil Jet Oil 11, were caused by exposure to the oil or any of its components’.^64^ For Plummer and his industry allies, aircraft lubricants were unlikely source of toxicity or illness.

Aircraft engineers defended existing air intake designs while also pursuing alternatives. ‘To me, as an engineer’, explained Ivor Williams, Chief Systems Engineer for British Aerospace, ‘it does not matter much whether it is a separate compressor driven by an electrical motor or a compressor driven by an engine. It will come to the same thing in the end, because it will have oil and bearings in it and they will be subject to failure. Indeed, aeroplanes have been like that for a long while’.^65^ For such industry engineers, all designs carried inherent risks and the bleed air design struck the right balance between safety and practicality. Despite this defence, evidence suggests that some manufacturers developed a safer alternative. Boeing Aircraft introduced a new air intake system for the Boeing 787 Dreamliner, which does not use bleed air to supply the cabin; instead, air intakes have been mounted along the root of the front wings, thereby reducing the risk of contamination.^66^

Despite a lack of clinical recognition for AS, concerned pilots, aircrews, researchers, and employee unions, have worked to build an evidence base and increase public awareness. Pilots offered testimony about how fume events affected their health. Former Cathay Pacific pilot Ben Holmes remembered: ‘I have had two really good doses of this stuff and it has brought my health to the point where I can barely function’.^67^ He recalled one instance of exposure when landing of a commercial airliner: ‘I was missing a lot of things’, he remembered, ‘and the airplane was just getting ahead of me. When an airplane gets ahead of you at 8 miles a minute, it's a long way ahead of you’.^68^ Holmes described how he began to feel ill after inhaling fumes in cockpit. ‘I was convulsing, having almost full fits; and these were coming in waves: so, one every ten or fifteen minutes. I thought I was having some sort of epileptic event’.^69^ Although he landed the plane safely with the help of his co-pilot and reported the event to his superiors, it was his feeling of responsibility to the passengers which motivated his testimony.

Material datasheets and toxicology reports have become important sources for AS proponents for understanding the health effects of pyrolyzed engine lubricant.^70^ University of British Columbia toxicologist, Chris van Netten, explained that in his opinion ‘there shouldn't be any in the aircraft. The tricreyl phosphates are meant to be in the engine and not in the aircraft itself. That certainly means that the oil has been burnt and enters the cabin, and that people are inhaling this material’.^71^ The lubricants have also been shown to contain toxic substances and according to the warning label on one type of jet oil: ‘Prolonged or repeated breathing of oil mist, or prolonged or repeated skin contact can cause nervous system effects’.^72^ Such expert testimony and datasheets provided important evidence for AS proponents and their allies.

Enhanced methods of data collection and analysis have been endorsed by AS proponents. The former senior advisor to the European Environmental Agency, David Gee, argued in 2019 that the Bradford Hill method could be applied to AS and help move it from ‘an observed association to a robust causal inference’. The method, developed in the 1960s by epidemiologist and statistician Austin Bradford Hill, set out nine principles which could be used to determine epidemiologic evidence of a cause and effect relationship.^73^ The method was successfully used to link tobacco smoke with lung cancer. Gee reasoned that ‘Bradford Hill would probably have assigned “fair” evidence for AS causality’.^74^ In turn, some toxicologists recommended the adoption of a standardized assessment protocol and a set of criteria to assist with clinical diagnosis of ‘potential AS’.^75^ Such proposals have pointed to clear pathways to help determine the cause of AS.

AS proponents have championed peer-reviewed research and aided in its production. They have worked independently and collaboratively to gather samples and support university-affiliated scientists. Former airline pilot and researcher Susan Michaelis began carrying an air testing device when she travelled on aircraft to capture data for analysis. Likewise, some former pilots have taken swab samples of cabin surfaces to track chemical residues. Although research affirming AS has appeared in peer-reviewed public health, medical and toxicology journals, this evidence has been met with strident industry opposition.^76^ For instance, in 2017, Michaelis co-published an article arguing that based on the evidence AS should be considered an occupational disease.^77^ The airline industry's Aerospace Medical Association countered that after ‘review of this paper, we see no evidence identifying such a relationship and given the controversial nature of the subject matter, wish to convey our concerns about the methodology of the study’. The physicians claimed that the study did not follow consistent quantitative analysis or meet the criteria for causation.^78^ In response, Michaelis and her co-authors published a defence of their work, refuting each point raised by industry physicians.^79^ Peer-reviewed journals have become sites of contestation over research methods and ultimately the recognition of AS.

AS proponents have worked closely with employee unions, such as Unite in Britain and Ireland, to achieve recognition and support.^80^ Through such collaboration, in March 2016 Unite demanded an independent public enquiry into the potential health effects of cabin air, and organized a legal case on behalf of sixty-one crew members.^81^ ‘The issue of toxic cabin air is so serious that our cabin crew members are likening it to the impact of asbestos in the building industry’, Unite's executive director for legal affairs, Howard Beckett, explained.^82^ Similarly, the European Cockpit Association released a position paper, arguing for increased air monitoring and the belief that ‘cabin air contamination by chemicals from the engine and/or hydraulic oil, is a known problem’.^83^ Such activism has helped to raise awareness of AS and inspired further investigations with the aim to formally recognize AS.

The media has remained an important ally of AS proponents.^84^ Television producers, magazine editors and journalists have reported on the issue for decades and most of the coverage has been sympathetic to pilots and aircrews. Some headlines have roused public attention: ‘We’ll be looking for TOMBSTONES: A Boeing engineer's DEADLY warning about toxic cabin air’, reported *The Express*, while *BBC News* claimed that ‘Airlines face lawsuits over ‘toxic’ cabin air’.^85^ Investigative news programs, such as *60 Minutes*, have interviewed former pilots about AS and pressed airlines to respond.^86^ Such coverage has helped proponents to move discussions about AS into the public sphere.

Although national governments in the United States, United Kingdom, Germany and Australia, have convened expert groups since the late 1990s to examine AS, the recommendations have advocated further research rather than clinical recognition. In the United States, the National Research Council convened a committee in 2000 to study aircraft environmental control systems and sources of contamination. The report, released two years later, showed that cabin air contamination occurred and proposed a series of recommendations, including improved air quality measurement.^87^ The report inspired the US Congress to pass legislation in 2003, requiring the Federal Aviation Administration to fund research into cabin air quality. Despite a promising start, staff working on the project were unable to secure cooperative agreements with airlines to carry air sampling devices.^88^

In Europe, the UK government commissioned studies on AS, but did not find a link between contaminated cabin air and illness. In 2007, the government directed an expert committee to review the literature and to assess the incidence of fume events. The committee found that fume events occurred in around 0.05% of flights, but evidence was insufficient to link them to illnesses.^89^ Later, in 2011, the UK government commissioned an air quality study in 100 flights and found no evidence of fume events.^90^ Following this study, a House of Lords expert committee examined the literature and concluded that while cabin air could become contaminated ‘a toxic mechanism was an unlikely cause for the reported illnesses’.^91^ Similarly, in 2012, the Fraunhofer Gesellschaft in Germany was charged by the European Agency for Flight Safety to study cabin air quality. The resulting 2016 report found that organophosphates were present in the air but in such small amounts that the authors believed they were an unlikely source of illness. They advised the introduction of detailed classifications for cabin air contamination and further human exposure studies.^92^

Australia approached AS by focusing on the risks posed by specific aircraft. In 1999, the Australian government established a special committee to study the air quality of the BAe 146 aircraft.^93^ The final report set out a number of recommendations, including improved maintenance and air quality monitoring, the use of a modified air intake system, the installation of high grade air filters, a review into the hazards of lubricating oil and a comprehensive research study into health effects of exposure to aircraft cabin air on air crew and passengers’.^94^ Ten years later, the Australian Civil Aviation Safety Authority established an expert panel that argued that lubricating oils could contain chemicals known to produce ill effects but there was no association to a lasting illness.^95^

Debates over the recognition of AS appear far from settled. Although fume events have been reported by pilots and flight crews for decades, disagreement remains over whether such exposure can cause an illness. Proponents of AS, including many pilots, flight crews and their unions, have drawn on and contributed to the production of peer-reviewed studies appearing in toxicology, medical and public health journals. Opponents of AS, including aircraft manufacturers, airlines and their medical advisors have attacked the conclusions and methods of proponents, and cited the confidentiality of personnel files and the impact of contributing risk factors to rationalize their dissent.

Despite a growing body of peer-reviewed research supporting the recognition of AS, the science remains contested for many reasons. First, what constitutes sufficient evidence is unsettled, as the threshold for scientific rigor has increased since the 2000s and linked to costly replication studies.^96^ Second, since AS research is contentious, few scientists are willing to jeopardize their careers to pursue studies that might alienate industry partners or fail to attract external funding. Third, there is a lack of coordinated global leadership because few international organizations have the clout or resources to influence national policymaking or make resolving AS a priority. Finally, the uncertain impact of industry affiliation bias may have shaped how scientific evidence was evaluated and discussed, thereby inhibiting a collaborative approach to AS research.

The case of AS serves an important reminder of how science can become politicized when the stakes are high. For proponents, formal recognition of AS means acknowledgement of a decades-old health risk and occupational illness, which could facilitate claims for compensation and improved medical treatment. For opponents, it means the potential for litigation, tighter regulation and embarrassing headlines, as well as demands for improved cabin filtration and aircraft retrofitting or redesign. For policymakers, tasked with responding to the science of AS, the economic and political significance of aircraft manufacturing and the airline industry has inspired a cautious strategy, advocating further research over formal recognition. For the family of deceased air steward Matthew Bass, ongoing debates have provided little comfort and failed to resolve what their son may have risked while doing the job he loved.

## Notes

1 Kate Leahy, ‘“There are hundreds of sick crew”: is toxic air on planes making frequent flyers ill?’, *The Guardian*, https://www.theguardian.com/science/2017/aug/19/sick-crew-toxic-air-planes-frequent-flyers-ill (last accessed 1 August 2020).

2 Dianne Apen-Sadler, ‘Coroner rules British Airways steward, 34, died due to cardiac arrest after drinking four times the drink-drive limit despite parents’ fear had been poisoned by toxic cabin air’, *The Daily Mail*, https://www.dailymail.co.uk/news/article-5674715/British-Airways-steward-died-drinking-four-times-limit-not-toxic-cabin-air-coroner-rules.html (last viewed 1 August 2020).

3 Debate pack: Cabin air safety and aerotoxic syndrome, Number CDP 2016-0068, 15 March 2016, 7.

4 ‘Why Matt Died’, http://mattbass.co.uk/why-matt-died/ (last accessed 1 August 2020).

5 Gerard Hageman, et al., ‘Aerotoxic Syndrome, Discussion of Possible Diagnostic Criteria’, *Clin Toxicol (Phila)*, 58, 5 (May 2020), 414–6; J. Burdon, ‘The “Aerotoxic Syndrome” – real condition or flight of fancy?’, *Australian & New Zealand Journal of Health, Safety and Environment*, 27 (2011), 201–4; W. Winder and S. Michaelis, ‘Crew Effects from Toxic Exposures on Aircraft’, in *Handbook of Environmental Chemistry* 4, H. (Springer-Verlag, Berlin/Heidelberg, 2005), 223–42; C. V. Howard, S. Michaelis, and A. Watterson, ‘The Aetiology of “Aerotoxic Syndrome” – A Toxico-pathological Viewpoint’, *Open Acc J of Toxicol*, 1 (2017), 1–3; S. Michaelis, J. Burdon, and C. Howard, ‘Aerotoxic Syndrome: A New Occupational Disease?’, *Public Health Panorama*, 3 (2017), 198–211; A. Harper, ‘A Survey of Health Effects in Aircrew Exposed to Airborne Contaminants’, *Journal of Occupational Health & Safety, Australia & New Zealand*, 21 (2005), 433–9; D. Gee, ‘Associations and Causation: A Bradford Hill Approach to Aerotoxic Syndrome’, *Journal of Health and Pollution*, 9, 24 (December 2019), S1–142.

6 C. E. Furlong, ‘Genetic Variability in the Cytochrome P450-paraoxonase 1 (PON1) Pathway for Detoxication of Organophosphorus Compounds’, *J Biochem Mol Toxicol*, 21, 4 (2007), 197–205; C. E. Furlong, T. B. Cole, R. J. Richter, N. K. Yee, L. G. Costa, and M. J. MacCross, ‘Biomarkers for Exposure and Sensitivity to Organophosphorus (OP) Compounds’, Paper presented at the Contaminated Air Protection Conference (2005).

7 Brinda Sarathy, Vivien Hamilton, and Janet Farrell Brodie (eds), *Inevitably Toxic: Historical Perspectives on Contamination, Exposure, and Expertise* (University of Pittsburgh Press, 2019); Richard J. Evans, ‘History, Memory, and the Law: The Historian as Expert Witness’, *History and Theory*, 41, 3 (October 2002), 326–45.

8 Allan M. Brandt, *The Cigarette Century: The Rise, Fall, and Deadly Persistence of the Product That Defined America* (Basic Books, 2009). See also Sarah A Felknor, Paul A Schulte, Teresa M Schnorr, Regina Pana-Cryan, and John Howard, ‘Burden, Need and Impact: An Evidence-Based Method to Identify Worker Safety and Health Research Priorities’, *Annals of Work Exposures and Health*, 63, 4 (May 2019), 375–85.

9 David Rosner and Gerald Markowitz, *Deadly Dust* (Princeton University Press, 1994); Michelle Follette Turk, *A History of Occupational Health and Safety: From 1905 to the Present* (University of Nevada Press, 2020); Brett Walker and William Cronon, *Toxic Archipelago: A History of Industrial Disease in Japan* (University of Washington Press, 2011); Christopher C. Sellers, *Hazards of the Job: From Industrial Disease to Environmental Health Science* (University of North Carolina Press, 1999); Alan Derickson, *Black Lung: Anatomy of a Public Health Disaster* (Cornell University Press, 2014); Claudia Clark, *Radium Girls: Women and Industrial Health Reform, 1910–1935* (University of North Carolina Press, 1997); Allard E. Dembe, *Occupation and Disease: How Social Factors Affect the Conception of Work-Related Disorders* (Yale University Press, 1996); Susan L. Smith, *Toxic Exposures: Mustard Gas and the Health Consequences of World War II in the United States* (Rutgers University Press, 2019). Books written by AS proponents, include Porter Lafayette, *Fume Event: Aviation's Biggest Lie* (LuLu, 2017); John Hoyte, *Aerotoxic Syndrome: Aviation's Darkest Secret* (Pilot Press, 2014); Bearnairdine Beaumont, *The Air I Breathe – It's Classified* (Writersworld, 2015).

10 Christoph Gradmann and Jonathan Simon (eds), *Evaluating and Standardizing Therapeutic Agents, 1890–1950* (Palgrave Macmillan, 2010); Roger Cooter and Claudia Stein, *Writing History in the Age of Biomedicine* (Yale University Press, 2013); Jeremy A. Greene, Flurin Condrau, and Elizabeth Siegel Watkins (eds), *Therapeutic Revolutions: Pharmaceuticals and Social Change in the Twentieth Century* (University of Chicago Press, 2016).

11 Landon R. Y. Storrs, *Civilizing Capitalism: The National Consumers’ League, Women's Activism, and Labor Standards in the New Deal Era* (University of North Carolina Press, 2000); Rosemary Feurer and Chad Pearson (eds), *Against Labor: How U.S. Employers Organized to Defeat Union Activism* (University of Illinois Press, 2017); Peter Cole, *Dockworker Power: Race and Activism in Durban and the San Francisco Bay Area* (University of Illinois Press, 2018); Matthew Hild and Keri Leigh Merritt (eds), *Reconsidering Southern Labor History: Race, Class, and Power* (University Press of Florida, 2020).

12 John Duffy, *The Sanitarians: A History of American Public Health* (University of Illinois Press, 1992); Judith W. Leavitt and Ronald L. Numbers (eds), S*ickness and Health in America: Readings in the History of Medicine and Public Health* (University of Wisconsin Press, 1997); George Rosen, *A History of Public Health* (Johns Hopkins University Press, 2015); Randall M. Packard, *A History of Global Health: Interventions into the Lives of Other Peoples* (Johns Hopkins University Press, 2016).

13 Victoria Vantoch, *The Jet Sex: Airline Stewardesses and the Making of an American Icon* (University of Pennsylvania Press, 2013); William Stadiem, *Jet Set: The People, the Planes, the Glamour, and the Romance in Aviation's Glory Years* (Ballantine Books, 2014).

14 *The Airliner Cabin Environment and the Health of Passengers and Crew*, Committee on Air Quality in Passenger Cabins of Commercial Aircraft, Board on Environmental Studies and Toxicology (National Research Council, National Academies Press, 2002), 1.

15 *The Airliner Cabin Environment and the Health of Passengers and Crew*, 34.

16 Stewart Wilson, *Legends of the Air Boeing 707 Douglas DC-8 Vickers VC10* (Aerospace Publications, 1998).

17 *The Airliner Cabin Environment and the Health of Passengers and Crew*, 5; John Hoyte, *Aerotoxic Syndrome: Aviation's Darkest Secret* (Pilot Press, 2014).

18 Marc Houtzager, et al., ‘Characterisation of the Toxicity of Aviation Turbine Engine Oils after Pyrolysis (AVOIL) – Final Report’, European Aviation Safety Agency, February 16, 2017; Civil Aviation Authority, ‘Cabin Air Quality’, February 2004.

19 John P. Morgan, ‘The Jamaica Ginger Paralysis’, *JAMA*, 248 (1982), 1864–7.

20 Rachel Carson, *Silent Spring* (Boston, Mass.: Houghton Mifflin, 1962; London: Hamish Hamilton, 1963); *Reports of the Working Party: Toxic Chemicals in Agriculture* (London: HMSO, 1951); *Toxic Chemicals in Agriculture: Residues in Food* (London: HMSO, 1953); and *Toxic Chemicals in Agriculture: Risks to Wildlife* (London: HMSO, 1955).

21 B. Acohido, ‘Alaska airlines jet flew repeatedly with fouled air’, *The Seattle Times*, January 21, 2000; T. J. Clark, ‘British pilots overcome by fumes’, *The Observer*, April 22, 2001; *The Airliner Cabin Environment and the Health of Passengers and Crew*, 2; *Air Safety and Cabin Air Quality in the BAe 146 Aircraft* (2000), 13.

22 Tara Brown (Reporter), *60 Minutes* (Australia), December 3, 2013.

23 *Air Safety and Cabin Air Quality in the BAe 146 Aircraft* (2000), xiii.

24 *Air Safety and Cabin Air Quality in the BAe 146 Aircraft*, 11.

25 Senate Rural and Regional Affairs and Transport References Committee, ‘Air Safety and Cabin Air Quality in the BAe 146 Aircraft’, Parliament of the Commonwealth of Australia, October 2000, 19; Committee on Toxicity, ‘House of Commons Debate Pack: Cabin air safety and aerotoxic syndrome’, March 17, 2016; Susan Michaelis, ‘Health and Flight Safety Implications from Exposure to Contaminated Air in Aircraft’, PhD Thesis, Sydney, UNSW (2010); Susan Michaelis, ‘Contaminated Aircraft Cabin Air’, *Journal of Biological Physics and Chemistry*, 11 (2011), 132–45; J. Murawski and D. Supplee, ‘An Attempt to Characterize the Frequency, Health Impact, and Operational Costs of Oil in the Cabin and Flight Deck Supply Air on US Commercial Aircraft’, *J ASTM Int*, 5 (2008), 1–15; J. Anderson, ‘Sources of Onboard Fumes and Smoke Reported by U.S. Airlines’, *Aerospace*, 8 (2021), 122; M. Shehadi, B. Jones, and M. Hosni, ‘Characterization Of The Frequency And Nature Of Bleed Air Contamination Events In Commercial Aircraft’, *Indoor Air*, 26 (2015), 478–88.

26 Notably the BAe 146, the McDonnell Douglas 80 and 90 series, the Boeing 737, and the Airbus A300 and A320 series. See Air Safety and Cabin Air Quality in the BAe 146 Aircraft (2000), 15, 97.

27 G. A. Gutowski, R. N. Page, and M. D. Peterson, ‘Decontamination Program’, Boeing Airplane Company, Document D-14766-2, December 18, 1953, 1–3.

28 Gutowski, et al., ‘Decontamination Program’, 32.

29 Ted A. Loomis and Stephen Krop, ‘Medical Laboratories Special Report No. 61: Cabin Air Contamination in RB-4A Aircraft’, Chemical Corps Medical Laboratories, February 1955.

30 Loomis and Krop, ‘Medical Laboratories’, 44.

31 Loomis and Krop, ‘Medical Laboratories’, 40.

32 Loomis and Krop, ‘Medical Laboratories’.

33 Loomis and Krop, ‘Medical Laboratories’, 1.

34 Susan Lederer, *Subjected to Science* (Johns Hopkins University Press, 1998).

35 Loomis and Krop, ‘Medical Laboratories’, 7.

36 Smith, *Toxic Exposures*.

37 Loomis and Krop, ‘Medical Laboratories’, 11.

38 Colonel Wolfgang W. E. Samuel and R. Cargill Hall, *Silent Warriors, Incredible Courage: The Declassified Stories of Cold War Reconnaissance Flights and the Men Who Flew Them* (University Press of Mississippi, 2019).

39 CS-25.1309c and Occupational Safety and Health (OSH) Act, Public Law 91–516.

40 Sarah Milov, *The Cigarette: A Political History* (Harvard University Press, 2019); Allan M. Brandt, *The Cigarette Century: The Rise, Fall, and Deadly Persistence of the Product That Defined America* (Basic Books, 2009).

41 *The Airliner Cabin Environment and the Health of Passengers and Crew*, 4.

42 *The Airliner Cabin Environment and the Health of Passengers and Crew*, 6.

43 *Air Safety and Cabin Air Quality in the BAe 146 Aircraft*, 14–21; Winder and Michaelis, ‘Crew effects from toxic exposures’, 216; Christiaan van Netten, ‘Contaminated Air Protection Conference: Proceedings of a Conference, held at Imperial College, London, 20–21 April 2005’, *Aircraft Air Quality Incidents, Symptoms, Exposures And Possible Solutions* (University of New South Wales, Sydney, 2005); Expert Panel on Aircraft Air Quality, ‘Contamination of Aircraft Cabin Air by Bleed Air – A Review of the Evidence’, Australian Government, Civil Aviation Safety Authority (2009), 35.

44 Tim van Beveren (Reporter), ‘Toxic Airlines and the Aerotoxic Syndrome’, 9 March 2009, Markt TV.

45 Tim van Beveren, ‘Toxic Airlines’.

46 *The Airliner Cabin Environment and the Health of Passengers and Crew*, 18.

47 *Air Safety and Cabin Air Quality in the BAe 146 Aircraft*, 3, 20.

48 *Air Safety and Cabin Air Quality in the BAe 146 Aircraft*, 29.

49 *Submission 6*, Associate Professor Chris Winder, 12 in *Air Safety and Cabin Air Quality in the BAe 146 Aircraft* (2000), 30.

50 *Air Safety and Cabin Air Quality in the BAe 146 Aircraft*, 26.

51 Andrew C. Harper, ‘Corporate affiliation bias and BAe 146 aircraft: Senate report’, *Australian and New Zealand Journal of Public Health*, 25 (2001), 378.

52 *Air Safety and Cabin Air Quality in the BAe 146 Aircraft*, 4.

53 Brown, *60 Minutes*.

54 British Aerospace, Evidence, 10 April 2000, p 223 from *Air Safety and Cabin Air Quality in the BAe 146 Aircraft* (2000), 18.

55 Brown, *60 Minutes*.

56 Brown, *60 Minutes*.

57 Brown, *60 Minutes*.

58 Tim van Beveren, ‘Toxic Airlines’.

59 *The Airliner Cabin Environment and the Health of Passengers and Crew*, 119.

60 *Air Safety and Cabin Air Quality in the BAe 146 Aircraft*, 58.

61 *Air Safety and Cabin Air Quality in the BAe 146 Aircraft*, 19.

62 *Air Safety and Cabin Air Quality in the BAe 146 Aircraft*, 11.

63 *Air Safety and Cabin Air Quality in the BAe 146 Aircraft*, 43.

64 *Air Safety and Cabin Air Quality in the BAe 146 Aircraft*, 43.

65 Air Safety and Cabin Air Quality in the BAe 146 Aircraft (2000), 11.

66 House of Commons Debate Pack, Number CDP 2016-0068, 15 March 2016.

67 Brown, *60 Minutes*.

68 Brown, *60 Minutes*.

69 Brown, *60 Minutes*.

70 Brown, *60 Minutes*.

71 Tim van Beveren, ‘Toxic Airlines’.

72 Associate Professor Chris Winder, Evidence, 1 November 1999, 8. See also AFAP, Evidence, 1 February 2000, 113 in *Air Safety and Cabin Air Quality in the BAe 146 Aircraft* (2000), 45.

73 Austin Bradford Hill, ‘The Environment and Disease: Association or Causation?’, *Proceedings of the Royal Society of Medicine*, 58 (1965), 295–300.

74 David Gee, ‘Associations and Causation: A Bradford Hill Approach to Aerotoxic Syndrome’, in 2017 International Aircraft Cabin Air Conference, *Journal of Health and Pollution*, 9, 24 (December 2019), S1–142.

75 Gerard Hageman, Teake M. Pal, Jik Nihom, Sarah J Mackenzie Ross, and Martin van den Berg, ‘Aerotoxic Syndrome, Discussion of Possible Diagnostic Criteria’, *Clin Toxicol (Phila)*, 58, 5 (May 2020), 414–6.

76 C. Howard, ‘Inappropriate Use of Risk Assessment in Addressing Health Hazards Posed by Civil Aircraft Cabin Air’, *Open Access Journal of Toxicology*, 4 (2020), 65–71; Gerard Hageman, Teake M. Pal, Jik Nihom, et al., ‘Aerotoxic Syndrome, Discussion of Possible Diagnostic Criteria’, *Clinical Toxicology*, 58, 5 (2019), 1–3; C. Howard, S. Michaelis, and A. Watterson, ‘The Aetiology of ‘ Aerotoxic Syndrome ’ – A Toxico – Pathological Viewpoint’, *Open Access Journal of Toxicology*, 1, 5 (2017), 1–3; M. Shehadi, B. Jones, and M. Hosni, ‘Characterization of the Frequency and Nature of Bleed Air Contamination Events in Commercial Aircraft’, *Indoor Air*, 26 (2015), 478–88; Jeremy J. Ramsden, ‘Is there Such a Thing as Aerotoxic Syndrome?’, *Journal of Biological Physics and Chemistry*, 14 (2014), 113–6; Jeremy J. Ramsden, ‘Contaminated Aircraft Cabin Air: Aspects of Causation and Acceptable Risk’, *Journal of Biological Physics and Chemistry*, 12, 2 (2012), 56–68; A. V. Terry, ‘Functional Consequences of Repeated Organophosphate Exposure: Potential Non-Cholinergic Mechanisms’, *Pharmacology & Therapeutics*, 134 (2012), 355–65; S. Michaelis, ‘Contaminated Aircraft Cabin Air’, *Journal of Biological Physics and Chemistry*, 11 (2011), 132–45; Jeremy J. Ramsden, ‘The Scientific Adequacy of the Present State of Knowledge Concerning Neurotoxins in Aircraft Cabin Air’, *Journal of Biological Physics and Chemistry*, 11 (2011), 152–64; A. Harper, ‘A Survey of Health Effects in Aircrew Exposed to Airborne Contaminants’, *Journal of Occupational Health & Safety, Australia & New Zealand*, 21 (2005), 433–9; J. Murawski and D. Supplee, ‘An Attempt to Characterize the Frequency, Health Impact, and Operational Costs of Oil in the Cabin and Flight Deck Supply Air on US Commercial Aircraft’, *Journal of ASTM International*, 5 (2008), 1–15; R. Abeyratne, ‘Forensic Aspects of the Aerotoxic Syndrome’, *Med Law*, 21 (2002), 179–99.

77 Susan Michaelis, Jonathan Burdon, and C. Vyvyan Howard, ‘Aerotoxic Syndrome: A New Occupational Disease?’, *Public Health Panorama*, 3, 2 (2017), 141–356.

78 Aerospace Medical Association to *Public Health Panorama*, July 10, 2017.

79 S. Michaelis, J. Burdon, and C. Vyvyan Howard, ‘Response letter to the Aerospace Medical Association’, *Public Health Panorama* 17 August 2017.

80 ‘Debate pack: Cabin air safety and aerotoxic syndrome’, Number CDP 2016-0068, 15 March 2016, 4.

81 Unite, *Toxic air legal cases rise as MPs prepare to debate cabin air safet*y, 15 March 2016.

82 ‘Debate pack: Cabin air safety and aerotoxic syndrome’, Number CDP 2016-0068, 15 March 2016, 7.

83 ECA Position on Cabin Air Contamination, 2015, https://www.eurocockpit.be/positions-publications/eca-position-cabin-air-contamination (last viewed 2 August 2020).

84 Mike Powell, ‘Fume events: The toxic gases that may be harming aircrew and passengers’, *BBC New*s, 25 February 2020, https://www.bbc.co.uk/news/stories-51633897 (last viewed 15 August 2020); Natalie Paris, ‘Toxic cabin air campaigners call for detectors in planes: Mystery plane illness at Heathrow prompts calls for air quality warning systems in cabins’, *The Telegraph*, 29 Jan 2016, http://www.telegraph.co.uk/travel/news/Toxic-cabin-air-campaigners-call-for-detectors-in-planes/ (last viewed 1 August 2020); Hugh Morris, ‘Cabin crew sue Boeing over 'toxic cabin air': Four flight attendants blame contaminated cabin air on ill health after 'fume event' on board Boeing plane’, *The Telegraph*, 24 June 2015, http://www.telegraph.co.uk/travel/news/Cabin-crew-sue-Boeing-over-toxic-cabin-air/ (last viewed 1 August 2020); Peggy Hollinger, ‘UK Airlines face legal action because of quality of cabin air’, *Financial Times,* 8 June 2015, http://www.ft.com/cms/s/0/3009f22c-0def-11e5-8ce9-00144feabdc0.html#axzz42b5GRqDl (last viewed 1 August 2020); Andrew Gilligan, ‘Warning over toxic fumes in plane cabins: Coroner urges action to prevent deaths after warning toxic fumes in cabin air pose a health risk to frequent fliers and aircrew’, *The Telegraph*, 21 February 2015, http://www.telegraph.co.uk/news/aviation/11427509/Warning-over-toxic-fumes-in-plane-cabins.html (last viewed 1 August 2020).

85 Ted Jeory, ‘We’ll be looking for TOMBSTONES: A Boeing engineer's DEADLY warning about toxic cabin air’, *The Express*, 13 April 2014, https://www.express.co.uk/life-style/health/470109/We-ll-be-looking-for-TOMBSTONES-A-Boeing-engineer-s-DEADLY-warning-about-toxic-cabin-air (last viewed 8 August 2020); ‘Airlines face lawsuits over 'toxic' cabin air’, *BBC News*, https://www.bbc.co.uk/news/business-47740523 (last viewed 8 August 2020).

86 Brown, *60 Minutes*.

87 Committee on Air Quality in Passenger Cabins of Commercial Aircraft, Board on Environmental Studies and Toxicology, National Research Council, *The Airliner Cabin Environment and the Health of Passengers and Crew* (National Academies Press, 2002), xiv.

88 Hoyte, *Aerotoxic Syndrome*, 88.

89 Committee on toxicity of chemicals in food consumer products and the environment, 2007, 8 in *Contamination of aircraft cabin air by bleed air – a review of the evidence*, 54.

90 Aircraft Cabin Air Sampling Study; Part 1 of the Final Report, Report for DfT by the Institute of Environment and Health (Cranfield Ref No YE29016V), March 2011, vii.

91 House of Commons Debate Pack, Number CDP 2016-0068, 15 March 2016, 2.

92 Sven Schuchardt, Wolfgang Koch, and Wolfgang Rosenberger, ‘Cabin Air Quality – Quantitative Comparison of Volatile Air Contaminants at different Flight Phases during 177 Commercial Flights’, *Building and Environment*, 148 (2018), 148.

93 *Air Safety and Cabin Air Quality in the BAe 146 Aircraft*, x, xii.

94 *Air Safety and Cabin Air Quality in the BAe 146 Aircraft*, xvi.

95 Expert Panel on Aircraft Air Quality, ‘Contamination of aircraft cabin air by bleed air – a review of the evidence’, (2009), ix–x.

96 G. MacQueen, ‘What does it mean to have enough evidence?’, *J Psychiatry Neurosci*, 38, 1 (2013), 3–5; Editorial, ‘Nature at 150: Evidence in Pursuit of Truth’, *Nature*, November 9, 2019.

